# Honey bee behaviours within the hive: Insights from long-term video analysis

**DOI:** 10.1371/journal.pone.0247323

**Published:** 2021-03-17

**Authors:** Paul Siefert, Nastasya Buling, Bernd Grünewald

**Affiliations:** Institut für Bienenkunde, Polytechnische Gesellschaft Frankfurt am Main, Goethe-Universität, Frankfurt am Main, Germany; University of Alberta, CANADA

## Abstract

The combined behaviours of individuals within insect societies determine the survival and development of the colony. For the western honey bee (*Apis mellifera*), individual behaviours include nest building, foraging, storing and ripening food, nursing the brood, temperature regulation, hygiene and defence. However, the various behaviours inside the colony, especially within the cells, are hidden from sight, and until recently, were primarily described through texts and line drawings, which lack the dynamics of moving images. In this study, we provide a comprehensive source of online video material that offers a view of honey bee behaviour within comb cells, thereby providing a new mode of observation for the scientific community and the general public. We analysed long-term video recordings from longitudinally truncated cells, which allowed us to see sideways into the cells in the middle of a colony. Our qualitative study provides insight into worker behaviours, including the use of wax scales and existing nest material to remodel combs, storing pollen and nectar in cells, brood care and thermoregulation, and hygienic practices, such as cannibalism, grooming and surface cleaning. We reveal unique processes that have not been previously published, such as the rare mouth-to-mouth feeding by nurses to larvae as well as thermoregulation within cells containing the developing brood. With our unique video method, we are able to bring the processes of a fully functioning social insect colony into classrooms and homes, facilitating ecological awareness in modern times. We provide new details and images that will help scientists test their hypotheses on social behaviours. In addition, we encourage the non-commercial use of our material to educate beekeepers, the media and the public and, in turn, call attention to the general decline of insect biomass and diversity.

## Introduction

The survival, progress and homeostasis of a honey bee colony depend on the coordination of the individuals’ advantageous decisions. The complex social organization of honey bees, and other Hymenopteran insects, has been the subject of many studies (e.g. [1, 2]). This research, which has a history dating back centuries, has focused on the division of labour, such as comb construction, foraging, storing and ripening food, nursing the brood, temperature regulation and hygiene. However, as most of these behaviours are hidden from sight, no educational video material has existed until now.

Darwin took prominent steps to educate the public on bee behaviour, describing the remarkable comb-building activities of honey bees in his writings [3]. Similarly, in the early 19^th^ century, Huber observed nest building through a hive with glass sides, which opened like a bookshelf, allowing him to view the colony [4]. Huber’s observation hive was based on one conceptualized by de Réaumur, who had, with his novel construction, investigated the behaviour of honey bees through a glass surface [5]. These efforts to see inside the hive established one of the fundamental purposes of within-hive video observations.

Until the 21^st^ century, the available educational material regarding honey bee behaviour was almost exclusively confined to texts and illustrations. For example, studies using observation hives were cited in books, which described the following processes: the production and use of wax scales for comb construction [6, 7], the regression of foragers to nurses despite their advanced age [8, 9] and the varied dances bees use to communicate [10–13]. Now that online streaming platforms and digital recording technology enable the wide circulation of educational videos, honey bee behaviour should be made available in this form.

While various behaviours can be observed outside of comb cells, those within cells, such as brood care, are more challenging to observe, as vision is blocked by bees covering their respective cells. For example, in the early 20^th^ century, the development of larvae could only be examined by extracting larvae from the cells [14]. A solution to this issue was first proposed by Martin Lindauer [15]. By rotating comb strips 90°, and regulating the loss of temperature with a double layer of glass and providing only a small space for the hive to build, he induced the bees to raise brood in cells with a translucent cell wall. After observing these cells, Lindauer described the process of nursing the brood in writing, whereas 35 years later, an analogue video recording device was used for this purpose for the first time [16]. However, this video material never became publicly available, as the internet was in its infancy.

Along with developing new video techniques, which provided a side-view into cells, scientists discovered a previously unobserved behaviour: the active thermoregulation of honey bees within cells. Infrared cameras revealed that workers who showed very little movement were not resting but heating the comb from within the cells [17]. This finding complemented other important factors regarding thermoregulation that have been described extensively in the past [18–23].

In a recently published study [24], we combined our method of looking sideways into the cells with long-term digital recordings. We continuously recorded our observation hives’ brood areas, which resulted in detailed views of a wide range of honey bee behaviours and offspring development. While that study focused on the impact of neonicotinoids on nursing behaviour, we present here quantitative and qualitative analyses of social behaviour observed during these long-term recordings. These analyses include the quantification of brood cell visits and considerable video footage of worker behaviours, such as the creation and use of wax scales, deployment and uptake of pollen and nectar, brood care and inspection, thermoregulation, capping, cannibalism, grooming and surface cleaning. Our footage also shows the embryonic hatch and larval cocooning within the colony. Furthermore, we reveal several previously undescribed behaviours in detail, including comb remodelling, exceptional mouth-to-mouth feeding between a nurse and a larva, and pollen stamping by foragers. In addition, we further illuminate the method of water evaporation in brood cells. For the first time, we provide online, publicly accessible recordings of each of the aforementioned behaviours for educational purposes.

## Materials and methods

### Beehives and recording setup

**Fig 1:**
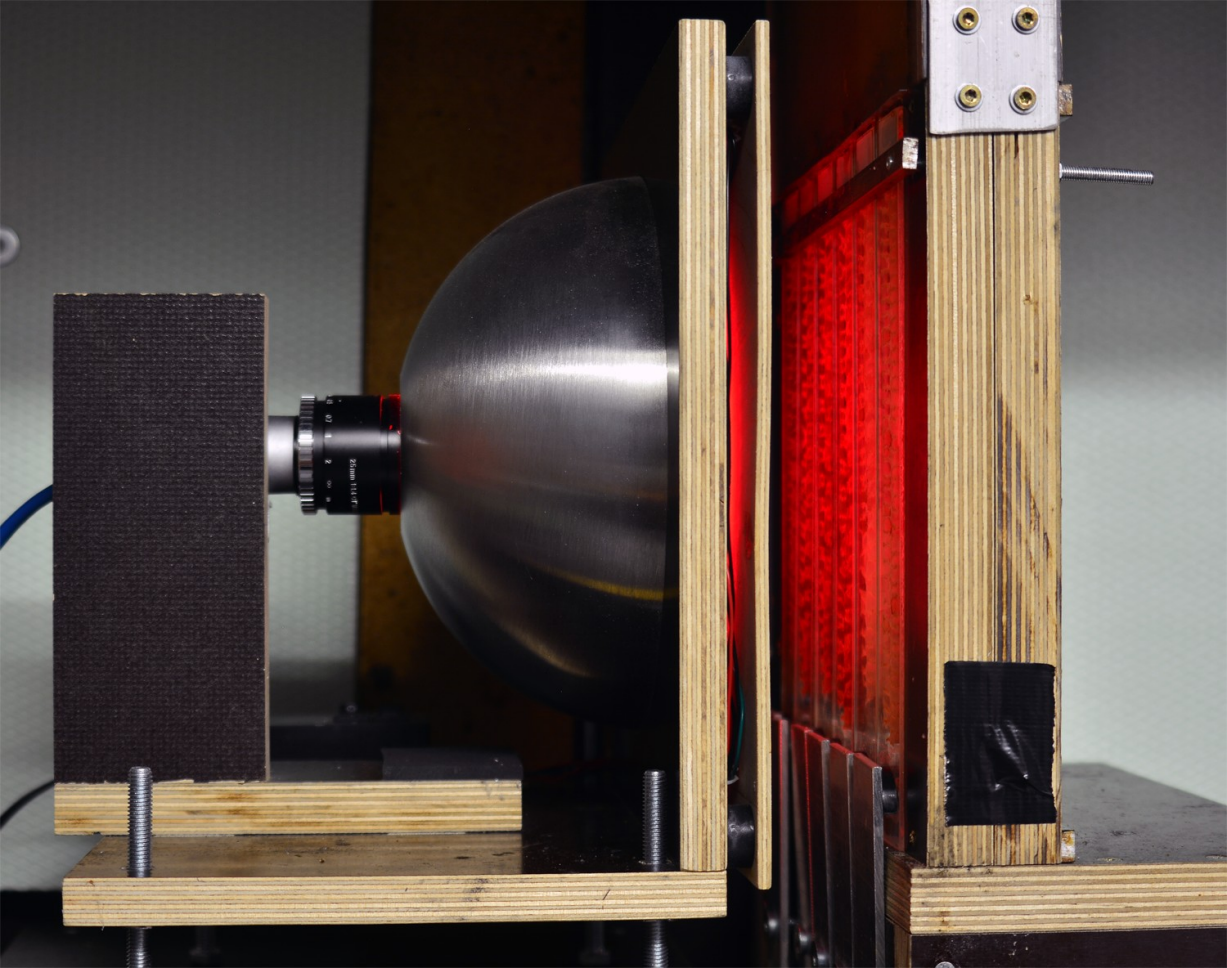
Macro video recording setup. The breeding area of an observation hive was illuminated by a dome-light emitting red light beyond honey bee colour vision. The dome was a 20 cm in diameter metal serving bowl, painted with matt white varnish from the inside, and had a large drilled hole in the top for the camera objective.

Experiments were based on the methodology presented in [24] (setup shown in Fig 1; for details see https://www.nature.com/articles/s41598-020-65425-y/figures/1). We used small observation hives, each with a population of approximately 3,000 individuals (300 g *Apis mellifera carnica*) and one queen. Bees and sister queens were taken from beehives and the on-site queen breeding programme of the Institut für Bienenkunde in Oberursel, respectively. In the designated brood area, the combs were turned 90°, enabling a view into the truncated cells through 4 mm of anti-reflective glass and the continuous recording of adult behaviours and offspring development therein. We either recorded the complete brood area using 5.3 MP cameras with 12.5 mm focal length lenses (camera PL-D725MU-T, PixeLINK, Ottawa with lens LM12HC, Kowa Optical Products Co., Ltd., Tokyo; or camera acA2500-60uc, Basler AG, Ahrensburg with lens TS1214-MP, Basler AG, Ahrensburg) or recorded in macro format with a 5 MP camera and a 25 mm focal length lens (camera acA2440-75um, Basler AG, Ahrensburg with lens TS2514-MP, Basler AG, Ahrensburg). While approximately 420 cells were observable with relatively low spatial and temporal resolution (1–3 frames per second) in the complete brood area recordings, macro recordings had a section of approximately 8 cells with high spatial and temporal resolution (25–30 frames per seconds). For illumination, we used red light-emitting dome-lights beyond the range of honey bee colour vision (λ_peak_ = 660 nm) with a diameter of 36 cm for overview recordings and 20 cm for macro recordings. StreamPix (version 6.3.0.155 and 7.4, NorPix Inc., Montreal) was the recording software used.

### Quantitative analysis of cell visits during worker development

We used the data and basic principles of the method described in [24] to examine the duration and depth of the workers’ visits within a brood cell in the current study. We projected videos (2D + time) into images (1D + time), concatenating the brightness of the cells’ centre pixels from the bottom to the entrance over time (see https://www.nature.com/articles/s41598-020-65425-y/figures/1). Bees were darker than the surrounding wax of the cell, and with a grey threshold, we were able to detect events. For each event, the algorithm described in [24] determined the total duration. The temporal resolution was one second in these experiments (recordings of all visible cells in the brood area).

## Results and discussion

### Egg movements and larval hatch

After oviposition (S1 Video), the egg remains motionless until the larvae hatch. As workers successively move as deeply as possible into the cells, eggs may be pushed down towards the cell base (S2 Video). Workers may move into the cells for preservation (clustering) or creation (direct incubation) of heat within the comb, and in the process, the worker and its antennae remain motionless. This observation is consistent with previous suggestions that a ‘tipping over’ is not part of the normal process of embryonal development [25] (cf. no tipping over in the S3 Video in [24]). Therefore, descending eggs reflect the probability of how often workers entered the cell for the purpose of thermoregulation. In some occasions, intense inspections can move the egg slightly (S3 Video). The larva’s hatch from its upright position is initiated by flexing and gradually increasing bending movements until the larva’s anterior end touches the surface of the wax. Afterwards, it does not pull itself erect and descends gradually to the cell base, or, in some cases, the side wall of the cell (S4 Video). During the hatch, the egg membranes are entirely dissolved [25]. The first feeding happens 95.2 ± 11.3 (mean ± SEM; n = 86) minutes after the larva hatches.

### Inspection, feeding and cocooning of larvae

**Fig 2:**
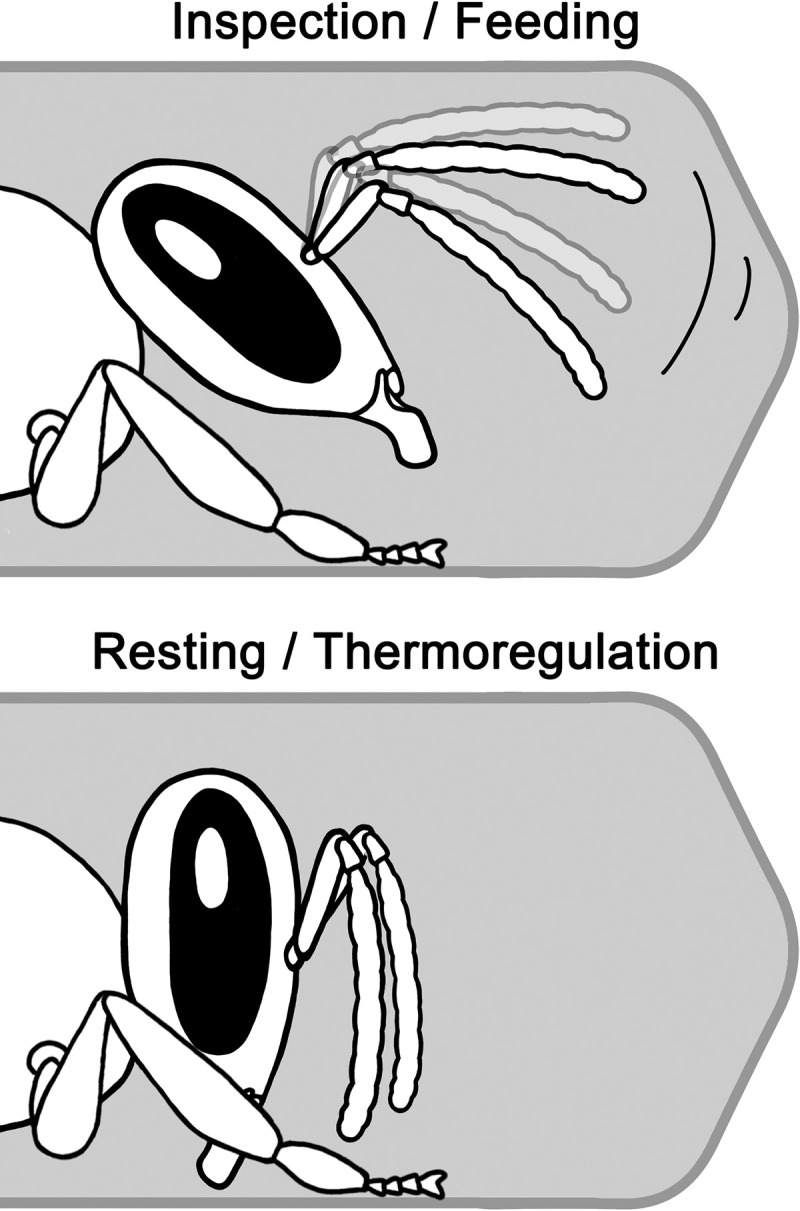
Differences in head alignment for different tasks when entering the cell. All drawings in this article are by Nastasya Buling.

Inspections include sensory information input and processing to determine the cell content, the location, status and age of brood, etc. The main characteristic of inspections is frequent antennal movement. During heating or resting behaviours, which are distinguished by the frequency of abdominal pumping movements [17], no antennal movement by the worker is present (Fig 2, S2 Video). Inspections that are not followed by other behaviours either occur for very short durations, in which the worker barely enters the cell, or for relatively long durations, which is more common in cells with very young larvae (S3 Video).

**Fig 3:**
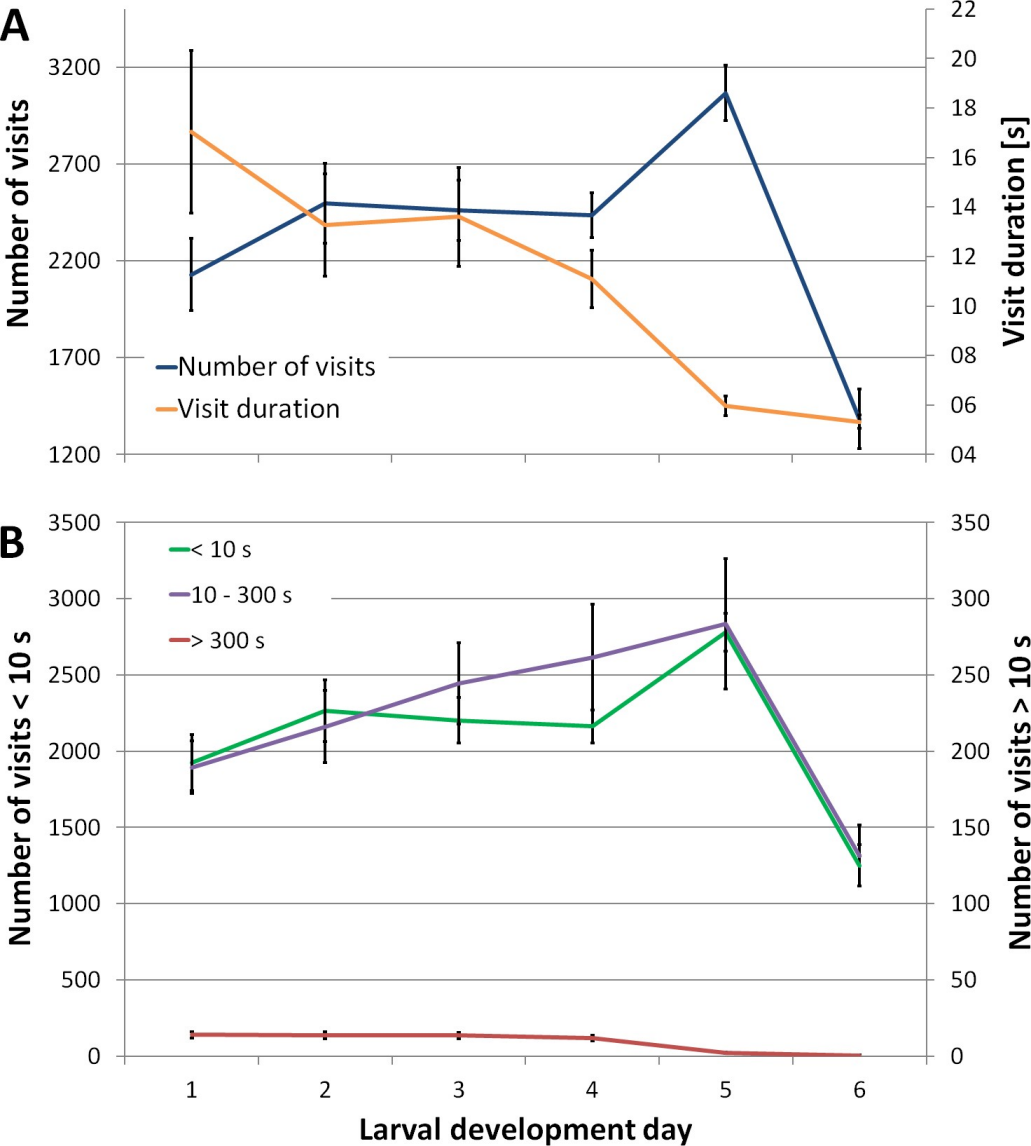
Mean number of visits and visit duration throughout larval development. Larval development was split into six larval development days. **A.** While the mean number of visits increased from the first to the fifth development day, the mean visit duration decreased by two thirds in this timespan. **B.** Visits of short duration (< 10 seconds) were tenfold higher than those combined of medium (10–300 seconds) and long (> 300 seconds) duration. The number of cells = 52 in 3 colonies recorded during 2016 and 2018.

In our quantitative analysis of cell visits in a cell with developing larvae, the mean number of short events (< 10 seconds) was tenfold higher than events of longer duration (Fig 3B). Throughout six larval development days (from the first to the last feeding), we detected 13,972 ± 617 events in the cell (mean ± SEM, n = 52 in 3 colonies). The number of events increased from 2,129 ± 186 during the first larval development day to 2497 ± 206 during the second day (Fig 3A). Similar numbers were counted on the third (2461 ± 154) and fourth (2436 ± 115) day. We observed the most events on the fifth (3065 ± 143) and the least on the sixth day (1383 ± 154). This prominent drop in events on the last larval development day was caused by the increasing chances that workers capped the cell. In contrast, the mean visit duration was highest during the first (17.0 ± 3.3 seconds) and lowest during the last larval development day (5.3 ± 0.3 seconds).

In cells containing a very young larva, inspections are often accompanied by longitudinal turns of the worker body. These turns reflect the worker’s attempt to locate the correct position beside the larva for food provision since covering the larva’s stigmata reduces its chance of survival. Between 10.7 and 62.8 percent of a feeding visit’s duration is spent on the inspection prior to food provision (n = 116). On larval development days 1–6, starting with the first feeding and ending with the last, inspections were 38.0 ± .9 (n = 116), 31.2 ± .6 (n = 115), 26.9 ± .6 (n = 110), 25.9 ± .4 (n = 97), 29.4 ± .7 (n = 68) and 28.2 ± 1.4 (n = 34) percent of the feeding visit duration, respectively (mean ± SEM; 8 colonies from May–June 2018).

Feedings are always preceded by an inspection, during which the worker shows strong antennal movements and directs its mouthparts and antennal tips towards the larva. After the inspection, the worker starts vibrating with its mandibles while gradually approaching the larva. During food provision, all tagmata usually remain motionless, while the antennae continue to move slightly. While food has to be carefully positioned for young larvae (S5 Video), workers can deposit food at any part of the surrounding cell walls near a larva three days or older. We observed most of the larval movement after feeding events, which indicates the intention of the larva to reach the freshly provided food. For example, larvae that receive a mouth-to-mouth feeding do not move after food provision (S6 Video). However, workers do not provide food exclusively close to or in the mouth of the larva, as it was suggested some time ago [14]. Furthermore, we never observed food provision prior to the hatching of the larva, as described elsewhere [16]. According to [24], feeding visits (including inspections) last 122.0 ± 10.4, 118.1 ± 3.3, 133.0 ± 4.8, 122.4 ± 2.8, 89.3 ± 2.4, 79.8 ± 2.8 seconds on larval development days 1–6, respectively (mean ± SEM; n = 4). The prominent decrease between days 4 and 5 may accompany the switch from ‘worker jelly’ to ‘modified worker jelly’ as has been previously reported [26, 27].

After the larva receives its last feeding, cocooning begins with tapping movements of the larva’s anterior end, where the silk glands are located (S7 Video). This movement initiates the transition from transversal to longitudinal turns (from here on: ‘somersaults’) within the cell. We measured 57.1 ± 1.5 (mean ± SEM, n = 17) somersaults during worker cocooning between the 31^st^ of May and the 14^th^ of July 2016 in 3 colonies. Cocooning took 32.2 ± 0.5 hours to complete and 34.1 ± 0.7 minutes were needed for one somersault. Compared to previous literature [28], we report approximately double the number of somersaults (27–37) and half the time needed for one (52 minutes) during cocooning.

### Comb construction and cell capping

Wax used for comb building can be found in two forms between the worker’s mandibles: firstly, as transparent wax scales, and secondly, as a non-transparent string-like form that is created from existing wax within the colony. In our observations, the latter has predominantly been seen on urgent occasions, such as quickly fixing the combs to the adjacent glass in the aperture shortly after the setup of the colonies. However, the use of wax strings can often be observed later in the colony’s development. Since the remodelling of combs includes individuals with undeveloped wax glands, it enables quick shifts in division of labour. To create a string of wax, the worker moves its head quickly back and forth, similar to a pecking bird, while the string between the mandibles is extended (S8 Video). The wax strings can be several millimetres in length and are extended below the caput and thorax. Long strings are folded for transportation using the prothoracic legs and the mandibles. To retrieve a wax scale from the intersternal pockets, the worker uses the basitarsal brushes of the hind leg [6, 7]. Retrieving the scale from the pocket takes about five seconds. The subsequent transportation of the scales to the mouthparts with the same leg takes just 400 ms (S9 Video). The use of wax scales and wax strings for building is the reverse of the process of wax string extension described above, including the rapid head and mandible movements. During building activity, workers move frequently within the cell, either back and forth or in longitudinal turns. Furthermore, frequent antennal and head movement is present. During cell capping, the worker frequently inserts its antennae into the closing hole of the cell as it is capped and puts its front tarsi onto the extended rim. We presume that the worker does so to measure the thickness of the cap. Capping is carefully adjusted to the larva’s developmental state, and cocooning starts before the cell is completely closed (S10 Video).

### Nectar and pollen storage

For storing nectar and honey, workers crawl ventrally upwards into the cell [29]. The food is then regurgitated from the worker’s stomach to the upper cell wall and spread by periodic half-circular movements (S11 Video). If the cell already contains liquid food, the mandibles dip into it. For the entire duration of the process, the proboscis remains folded and the mandibles are held open. Since food adheres to the upper cell wall and is pulled downwards by gravity, the cell can be evenly filled without the worker targeting the lower half of the cell. Liquid nourishment from the filled cell is taken up by the proboscis, an act that is possible regardless of the worker’s alignment to the cell.

**Fig 4:**
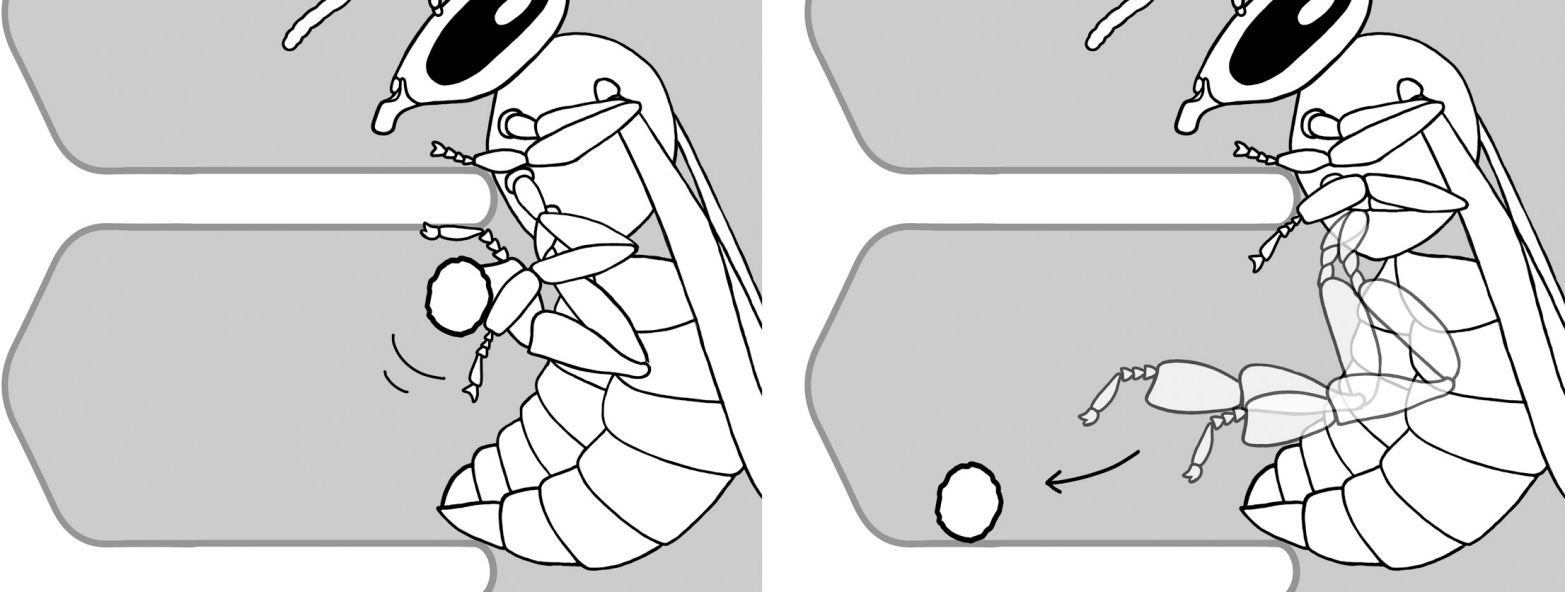
Pollen unloading into the cell.

Once a cell has been inspected and found suitable for pollen storage, the forager uses its prothoracic legs to hold on to the lower cell wall next to the inspected cell (Fig 4). The forager clasps the upper wall with its metathoracic legs while placing its bent abdomen on the lower wall of the suitable cell (S12 and S13 Videos). We did not observe workers thrust their metathoracic legs down into the cell and hang freely within it, as earlier reports state [30]. Instead, the corbiculae (pollen baskets) that contain the pollen are positioned at the cell entrance, and the mid legs remain free. The worker then uses the mesothoracic legs for a couple of slow brushes along the outer side of the hind legs, starting from the upper end of the tibia and then moving downward from the pollen mass to the corbicular surface. After the pollen load falls into the cell, the worker cleans any pollen remaining on the mid or hind legs in a similar fashion but with quicker movements. The worker then holds onto the upper cell wall with the pro- and mesothoracic legs to rub its metathoracic legs together, freeing them from small bits of pollen. The pollen that is now lying within the cell is then pushed further into the cell with several quick movements of the tarsi of the metathoracic legs. This process of leg cleaning and pollen pushing is repeated several times until the legs are free from remaining pollen. The forager then removes its legs and abdomen from the cell. Afterwards, younger bees nearby push the pollen further towards the base of the cell with closed mandibles and upward flicking movements of the head. On one occasion, this was done by the same forager re-entering the cell after pollen unloading (S12 Video). Packed pollen at the cell base is broken up and incorporated into the mass. During this process, the mass can be moisturized by the addition of saliva, nectar and honey [30] to create bee bread (S13 Video).

### Thermoregulation

As long as there is brood within a colony, honey bees maintain the surrounding brood area temperature at 33–36°C [17]. To avoid abnormalities in either the brood or in emerging adults when this range is exceeded [31], honey bees rely on various behaviours to regulate temperature. The process of heating the brood area includes the clustering of individual workers, the generation of metabolic heat and direct incubation (in which workers warm their thoraces through muscle contractions), while the process of cooling includes the dispersal of individuals, fanning and water evaporation (reviewed in [32]).

In our experiments, up to six observation hives were located at a distance of 3–5 metres to heat emitting radiators [24]. However, one additional hive, used for macro recordings, was right above the heat source. Consequently, most hives had a lower surrounding room temperature (~28°C; for details, see the discussion in [24]) than the one above the radiator (temperature not determined). In hives distant from the heat source, workers occupying the cell were detected motionless for up to 90 minutes. In hives directly above the radiator, the secretion of clear fluid drops into the cell was observed. Therefore, the workers’ immobility likely functions to actively generate heat via thoracic muscle vibration [20, 33, 34] or passively preserve heat via the clustering [22, 35] of resting bees throughout the combs. Bees showed no antennal movements during this period (S2 Video). Resting bees can be distinguished from those that generate heat by investigating their abdominal movements. While resting workers exhibit discontinuous and interrupted abdominal pumping movements, those of heat-generating workers are continuous and rapid [17]. Interestingly, we observed short- and long-term cell occupations without seeing antennal movements in cells with various stages of developing brood. During the process, the workers often moved successively deeper into the cell, an indicator of either active or passive thermoregulating behaviour. Until now, we have had no digital recordings of active comb heating via a crouched posture over the closed cells [23].

If the colony’s temperature exceeds tolerance levels, honey bees disperse across the combs and eventually leave the hive. At the hive entrance, workers start fanning, and foragers collect water to evaporate within the hive. This active increase of air humidity may also ensure offspring development; however, the brood nest has a relative humidity of only 30–50% [36], which is usually reached by the evaporation of stored nectar [37]. To avoid overheating, bees transfer droplets of clear fluid, mostly onto the upper walls of brood cells (S14 Video) and may spread it in a way that is similar to depositing nectar into cells (see below). In 14 observations (from the transfer of the first droplet until no droplets remained in the cell), only one droplet was left to evaporate, while all others were eventually taken up by workers. As evaporative cooling is a dynamic process, droplets can increase and shrink in size within minutes. However, in 9 of 14 observations of two hives, bees entered the cell once for both droplet transfer and uptake. In 11 of 14 cases, the cell was visited once for droplet transfer (mean ± SEM = 1.6 ± 0.4), and in 12 of 14 cases, the cell was visited once for droplet uptake (mean ± SEM = 1.0 ± 0.1). The time between visits for the deposit or uptake of fluids was 29.0 ± 4.7 minutes. However, a subsequent worker may take up some or all of the fluid just seconds after it is deposited (S14 Video). Droplet transfer took 19.3 ± 3.5 seconds, and uptake took 61.7 ± 11.7 seconds. If overheating is sudden (e.g. artificially generated by radiation of a 100-watt bulb or infrared radiator), workers use nectar until water foragers arrive [38]. Since the composition of the fluids in our videos is undetermined, the evaporative cooling may happen with nectar and/or water. However, the surrounding room temperature was stable, which supports the notion that water was used. Cooling may also occur when the worker holds a droplet between its tongue and its head while repeatedly folding and unfolding its tongue. While this strategy is used primarily for cooling, it is sometimes, though not always [38], used for ripening honey [39]. Unfortunately, we have no video material of this process at this time.

### Hygiene and cannibalism

Hygienic behaviour includes the removal and prevention of mould, fungi and parasites that endanger the colony’s survival. Workers exhibit a set of hygienic behaviours, such as cannibalism or cleaning themselves (auto-grooming), other nest mates (allo-grooming), surfaces (the ‘rocking movement’), cells, or larvae.

Cannibalism is an effective way for the colony to recycle proteins and prevent mould and fungi from growing on deceased offspring. However, in case of maldevelopment or diploid drones [40], offspring can be cannibalized by workers in every stage of their development (S15 Video), except during the last 72 hours when the cuticula hardens. In our observations, cannibalisation usually occurred without a visible cause (e.g. offspring turning dark), suggesting that workers perceive chemical information to identify diseased, deceased, parasitized or maldeveloped offspring [41]. We observed that larvae can move until and–to some degree–during cannibalization. We primarily observed cannibalism in the visible cells during the first couple of days of the experiment and more often during July and August than in May and June. This suggests that, with a decreasing brood, active workers prefer the inner cells for offspring development until they are eventually occupied. In such cases, young larvae can be cannibalized to enhance the survival probabilities of the older ones [42]. Interestingly, we very rarely observed cannibalisation of the eggs. Like the honey bee offspring, *Varroa destructor* mites can be consumed by workers when the cuticula is not hardened as in the female proto- or deutonymph or in the male. We observed mite consumption in one cell from which a new worker had just emerged (with two adult female mites) and, to our surprise, found very different reactions from the two workers that subsequently entered the cell. While the first one exclusively removed the mite’s defecations, the second one, which entered just a couple of minutes later, leaped forward with spread mandibles after touching the deutonymph with its antenna (S16 Video). Both mites were then consumed. When the mite’s cuticula is hardened, a grooming worker can still injure the shell or remove the legs with its mandibles to control *Varroa* infestation [43].

Worker bees perform a special behaviour to invite other nest mates for grooming. The ‘grooming invitation dance’ involves quick self-cleaning movements with the legs and waggling and bending of the body [44–46]. Auto- and allo-grooming efforts amongst *Varroa destructor* and *Tropilaelaps clareae* vary between *Apis* species [47], and social grooming is positively correlated to the degree of tracheal mite (*Acarapis woodi*) infection [48]. We had only a small chance of observing grooming invitation dances with our side-view vision system, but we offer a glimpse into allo-grooming behaviour in our online video material (S17 Video).

**Fig 5:**
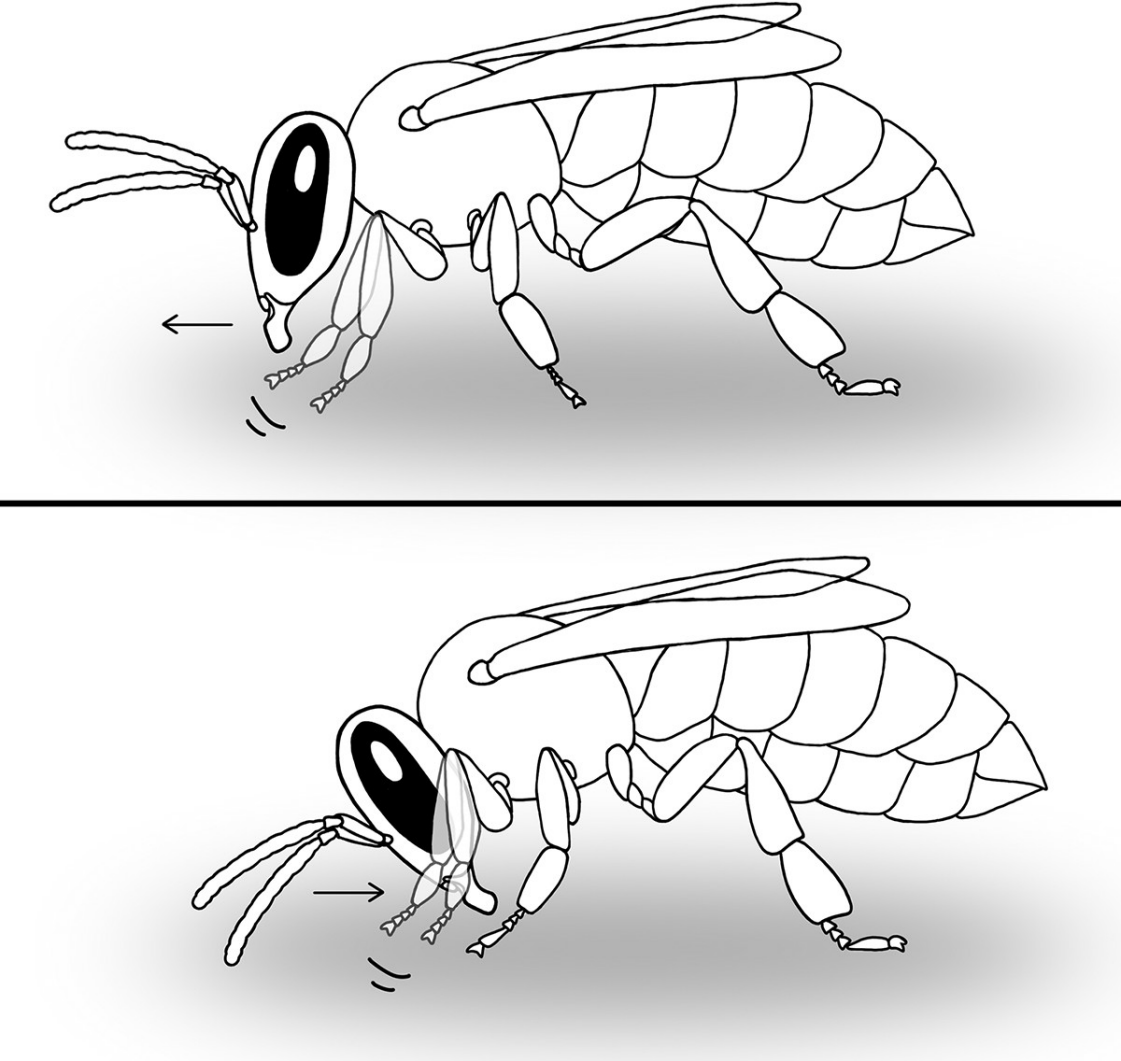
The ‘rocking movement’ for cleaning surfaces.

The mechanical cleansing of surfaces within a hive is also known as the ‘rocking movement’ [49], in which the worker’s mandibles and the tarsi of their front legs are used as scrapers. The worker sweeps the surface with quick repeating movements of its front legs and towards its mouthparts, while slowly leaning forwards. It then quickly resumes its original position, and the process is repeated several times over the same area. During this forward-leaning movement, the orientation of the mandibles shifts from a maximum posterior to an anterior position (Fig 5; S18 Video).

We observed 186 fluid uptakes by workers in 54 uncapped cells containing larvae. Compared to 5,363 feeding events in the same cells, the uptake of fluids is relatively rare and statistically occurs 3.5 ± 0.6 times (mean ± SEM) during larval development. However, in 19 of the 54 cells, this behaviour was absent, and 6 cells were cleaned between 11 and 20 times. Based on our observations, fluid uptakes are not linked to a specific time during development, and we did not see such cleaning behaviour on a regular basis or right before capping.

## Conclusions

While observations of honey bee behaviours date back centuries, the visualisations of many within-hive behaviours exist only in line drawings that are limited to representations of certain key moments. In this study, we present known and previously unknown honey bee behaviours in high-resolution videos, accessible online and free to the public. These videos may be used for further research or to educate beekeepers and public audiences. Our material may also help raise awareness of the general decline of flying insect biomass, insect biodiversity and the debate regarding the pollinator crisis [50]. We observed unique behaviours not previously described, such as mouth-to-mouth feeding of larvae or heat preservation/generation in cells containing offspring. The teaching of honey bee worker behaviour may contribute to an enhanced fascination with socially living insects. We therefore encourage teachers, scientists, journalists and others interested in insect behaviours to use our video footage for non-commercial educational and publication purposes.

## Supporting information

S1 Video Honey bee worker development: Oviposition. (MP4)

S2 Video Honey bee thermoregulation: Cell occupation. (MP4)

S3 Video Honey bee brood care: Short and long inspections. (MP4)

S4 Video Honey bee worker development: Larval hatch. (MP4)

S5 Video Honey bee brood care: Feeding of young larva. (MP4)

S6 Video Honey bee brood care: Mouth-to-mouth feeding. (MP4)

S7 Video Honey bee larval development: Cocooning. (MP4)

S8 Video Honey bee comb remodelling: Wax strings. (MP4)

S9 Video Honey bee comb construction: Wax scales. (MP4)

S10 Video Honey bee brood care: Capping. (MP4)

S11 Video Honey bee nutrition: Nectar storage and uptake. (MP4)

S12 Video Honey bee nutrition: Pollen storage. (MP4)

S13 Video Honey bee nutrition: Pollen unloading, packing and moisturisation. (MP4)

S14 Video Honey bee thermoregulation: Evaporative cooling. (MP4)

S15 Video Honey bee hygiene: Cannibalism. (MP4)

S16 Video Honey bee hygiene: *Varroa* consumption. (MP4)

S17 Video Honey bee hygiene: Allo-grooming. (MP4)

S18 Video Honey bee hygiene: The ‘rocking movement’. (MP4)
